# How well do questionnaires on symptoms in neck-shoulder disorders capture the experiences of those who suffer from neck-shoulder disorders? A content analysis of questionnaires and interviews

**DOI:** 10.1186/1471-2474-10-30

**Published:** 2009-03-09

**Authors:** Birgitta Wiitavaara, Martin Björklund, Christine Brulin, Mats Djupsjöbacka

**Affiliations:** 1 Department of Nursing, Umeå University, S-901 87 Umeå, Sweden; 2 Centre for Musculoskeletal Research, University of Gävle, S-801 76 Gävle, Sweden; 3 Centre for Musculoskeletal Research, University of Gävle, Box 7629, S-907 12 Umeå, Sweden; 4 Alfta Research Foundation, Box 94, S-822 22 Alfta, Sweden

## Abstract

**Background:**

Previous research has indicated neck-shoulder disorders to have a fluctuating course incorporating a variety of symptoms. These findings awoke our interest to make a comparison between symptoms experienced by people affected with the disorder and the content of questionnaires that assess pain and other symptoms in neck-shoulder disorders. Thus the aims of this study were: -to explore the symptoms experienced by people with non-specific neck-shoulder problems, as well as experiences of nuances and temporal variations (fluctuations) of symptoms; -to investigate which sources were used in the development of ten questionnaires for assessing pain and other symptoms in the neck-shoulder; -to analyse the item content of the questionnaires; -to analyse the correspondence between the item content of the questionnaires and the symptoms described by the informants.

**Methods:**

Content analysis of interviews with 40 people with non-specific neck-shoulder pain, and 10 questionnaires used to assess pain and other symptoms in neck-shoulder disorders.

**Results:**

The interviews revealed a variety of symptoms indicating a bodily, mental/cognitive, and emotional engagement, and more general and severe symptoms than are usually considered in neck-shoulder questionnaires. Taking all questionnaires together many of the symptoms were considered, but most questionnaires only included a few of them. The informants were able to distinguish fluctuation of symptoms, and a variety of different qualities which were not usually considered in the questionnaires. Only two questionnaires had made use of the opinions of affected people in the development.

**Conclusion:**

Few of the questionnaires had made use of the experiences of affected people in the development. The correspondence between the symptoms expressed by those affected and the content of the questionnaires was low. A variety of symptoms were expressed by the interviewees, and the participants were also able to distinguish nuances and fluctuations of symptoms. The present study points to the importance of other aspects than just pain and physical functioning as clinical trial outcome measures related to neck-shoulder disorders. To develop a condition-specific questionnaire, it is important to decide on the specific symptoms for the condition. Using the experiences of those affected, in combination with relevant research and professional knowledge, can enhance the validity of the questionnaires.

## Background

Musculoskeletal disorders are primarily a source of pain and human suffering, but they also have economic consequences, both for the individual and for society. Neck pain constitutes a large proportion of the musculoskeletal disorders. As much as 50% of the Swedish population is affected by neck pain at some point in life; this is comparable to international prevalence figures, which show a lifetime prevalence of 67–71% for neck pain [1]. The corresponding proportions for the point prevalence of neck pain varied between 12–22% [1]. Some recent studies also present similar results, showing a variation in the point prevalence between 21–43% for neck pain in Sweden and the Netherlands [2,3] and 20% for neck-shoulder pain in Japan [4]. The proportion of individuals suffering from chronic neck pain (duration > 12 weeks) has been estimated between 16–19% [2,5].

In general, the challenges related to musculoskeletal disorders are very complex since the knowledge on the pathophysiology is limited and a large number of potential risk factors have been identified [1]. In order to better understand and help people with neck pain, it is important to be able to measure this "pain and suffering" with valid questionnaires. One reasonable assumption for obtaining validity of questionnaires for any complex disorder or disease is that they are developed for the specific condition that they are intended to be used on. This, however, is not always the case, for example there are questionnaires for neck pain that are directly adapted from questionnaires for low back pain. Condition specific questionnaires would be valuable both in characterisation of the disorder and in evaluation of rehabilitative measures.

There are a number of ways to develop questionnaires for measuring pain and suffering. Generally, the questionnaires are based on theories or existing questionnaires, often from a professional perspective. It is important to take the experiences of those affected individual into consideration in the developmental process, which was also pointed out by several authors [6-9]. Consequently, an investigation of the experiences of sufferers could increase the face and content validity of the questionnaire via a higher relevance of the included items and adequacy of the questions for the intended use.

There is no consensus on how to investigate symptoms in neck-shoulder disorders neither in clinical practice nor in research. For instance, there is a wide variation in outcome measures in clinical trials for chronic pain, which makes comparison of treatment effects across studies difficult. The Initiative on Methods, Measurement, and Pain Assessment in Clinical Trials (IMMPACT) [10,11] recommends the following core outcome domains to consider when designing clinical trials: pain, physical functioning, emotional functioning, global improvement, satisfaction with treatment, symptoms and adverse events. The authors associated with IMMPACT further point out the value of also taking different quality and temporal aspects into account in order to create a fuller description of a patient's pain experience than can be gained from considering intensity alone. This fuller description would then make it easier to identify treatments that are effective for certain aspects of pain, and also enable better evaluations.

In previous studies, we explored health experiences among people with musculoskeletal problems [12-14]. Our findings indicated that chronic neck-shoulder disorders can be experienced as a state of constant discomfort with intermittent periods of increasing illness and peaks of consuming intensity. We also found that a variety of different symptoms were related to the course of the disorder [14]. This varied cluster of symptoms awoke our interest to make a comparison to symptoms included in neck-shoulder questionnaires.

The aims of this study were:

• to explore the symptoms experienced by people with non-specific neck-shoulder problems, as well as experiences of nuances and temporal variations (fluctuations) of symptoms

• to investigate which sources were used in the development of ten questionnaires for assessing pain and other symptoms in the neck-shoulder

• to analyse the item content of the questionnaires

• to analyse the correspondence between the item content of the questionnaires and the symptoms described by the participants.

## Methods

### Study design

Content analysis was performed in three steps – analysis of the interviews, analysis of the questionnaires, and a comparison of the results. First, interviews were conducted with a number of people experiencing non-specific neck-shoulder disorders. The interviews were reviewed to find out which symptoms the participants felt were related to their musculoskeletal problems. Thereafter, a number of questionnaires on symptoms in neck-shoulder disorders were chosen on the basis of a review of the literature [15], an additional literature search and discussions in the research group. Questionnaires that only presented items concerning disability or dysfunction were excluded. The papers describing the questionnaires were reviewed according to which sources were used in the development of the questionnaires, and the questionnaires were analysed with regard to the items measuring pain and other symptoms. Finally, a comparison between the content of the questionnaires and the content of the interviews was performed.

### Interviews – data collection

The informants constituted a convenience sample of 40 individuals, 25 women and 15 men (mean age 46.3 SD 7.8), with non-specific musculoskeletal problems in the neck-shoulder region. The participants were recruited via advertisements placed in a local paper in a medium-sized town in the middle of Sweden. Participants were primarily recruited to a larger project studying sensorimotor functions in chronic neck pain. The participants in that study were consecutively invited to participate in the present study until 40 participants were obtained. Everyone who was asked to participate in the interview study agreed to be interviewed, except one. The characteristics of the informants at the time of the interviews are presented in Table 1.

**Table 1:** Characteristics of informants

	n	%
Sex		
Women	25	62.5
Men	15	37.5

Last occupation		
Blue collar	21	52.5
White collar	15	37.5
Self employed	4	10

Occupational activity		
100%	31	77.5
75%	2	5
50%	4	10
25%	1	2.5
0%	2	5

Previous periods of sick leave for MSD		
Never	18	45
Shorter periods	12	30
> 3 months	10	25

Length of MSD problems		
< 5 years	3	7.5
5–9 years	4	10
10–14 years	5	12.5
15–19 years	5	12.5
> 20 years	14	35
Unable to specify	9	22.5

The interviews were semi-structured and made use of an interview guide, which had been developed on the basis of the results from two previous grounded theory studies exploring the health experiences of people with musculoskeletal disorders [12,13]. The focus of the interviews was to explore the symptoms and their temporal variations that the participants related to their musculoskeletal problems. All interviews followed the same interview guide, and follow-up questions were used to elicit the participants' answers. The interview guide included the following questions:

• Where are your problems sited?

• Please describe what you experience.

• Do you experience your problems all the time, or is it off and on?

• Are your problems the same all the time or do they change/have they changed?

• What do you experience when your problems get worse?

• How do you feel when your problems are at their worst?

• How did your problems start?

• How did you first notice these problems?

### Interviews – analysis

The interviews were analysed using content analysis [16] of the manifest content. First the interviews were read one by one, and all symptom descriptions that the participants related to their musculoskeletal problems were marked and a list of all symptoms was created. Then the interviews were re-read to check that nothing was missing in the symptom list. Next, the symptoms on the symptom list were collated into subcategories, which in turn were collated into categories and main categories. The categorisation was discussed within the research group as well as with two external specialists on musculoskeletal disorders. As a last step, the occurrence of each symptom in the sample was checked, and frequencies were calculated.

### Questionnaires – data collection

Questionnaires that presented items concerning pain and other symptoms were included. The choice of questionnaires was firstly based on a systematic review of papers published between 1966 and 2000 that considered standard scales for measurement of functional outcome for cervical pain or dysfunction [15]. This review presented the following questionnaires as standard neck pain scales:

• The Neck Disability Index (NDI) [17]

• The Neck Pain and Disability Scale (NPDS) [18,19]

• The Patient-Specific Functional Scale Self-Reports with Neck Dysfunction (PSFS) [20]

• The Northwick Park Neck Pain Questionnaire (NPQ) [21].

The review of Pietrobon and colleagues [15] also included the Copenhagen Neck Functional Disability Scale [22], which was excluded from our study since it only concerned neck dysfunction.

Secondly, a complementary search for papers on questionnaires which were published between 2000 and March 2007 was performed on the PubMed database using the keywords: neck pain scale; neck pain and outcome measures; neck pain and questionnaire. The following scales were found and added to the analysis:

• The Bournemouth Questionnaire (BQ) [23]

• The Cervical Spine Outcome Questionnaire (CSOQ) [24]

• The Core Neck Pain Questionnaire (CNPQ) [25]

• The Extended Aberdeen Back Pain Scale (EABPS) (neck, shoulder, low back) [26].

Finally, two more questionnaires were added, as they were judged to be relevant:

• The Profile Fitness Mapping questionnaires (PFM) (Björklund, Hamberg, Heiden & Barnekow-Bergkvist, In preparation)

• The Standardised Nordic Questionnaires (SNQ) [27].

The PFM questionnaire is region-specific and intended for assessment of symptoms and functional limitations in neck pain patients. The PFM was judged as relevant for this study, as the constructors of the scales made use of the experiences of people affected with chronic musculoskeletal problems in the development of the questionnaire. (Björklund et al. In preparation). Recently, a similar questionnaire intended for use among people with low back pain was tested for reliability and validity with good results [28]. The SNQ was added as it is often used in relation to musculoskeletal disorders. The SNQ consists of several different parts directed to different body regions. In the analysis in the present study the "Trouble with the locomotive organs" and "Questionnaire about neck and shoulder trouble" sections were chosen. In total, ten questionnaires were selected for analysis.

### Questionnaires – analysis

As a first step, the papers presenting the different questionnaires were reviewed for data on sources used in the development of the questionnaires and the items included. Secondly, all items regarding pain and other symptoms included in the questionnaires were listed, and sorted into different categories according to content. Items concerning disability or dysfunction with no connection to musculoskeletal pain were excluded. Next, the different aspects of descriptions of pain and other symptoms in the items, along with the response scales, were analysed. Various aspects of fluctuations (such as different time perspectives) and nuances of symptoms were considered in this analysis. The different aspects of pain were then categorised as they were addressed by the different questionnaires. Next, all symptoms included in the questionnaires were categorised with respect to engagement. Finally, the results of the analyses of the interviews and the questionnaires were compared for correspondence.

### Ethical considerations

The study was approved by the Regional Ethical Review Board in Uppsala (# 2006-013), Sweden. The participants received information orally and via an introductory letter about the purpose and procedure of the study, about guaranteed confidentiality, and that both participation and the choice of which experiences to communicate were voluntary.

## Results

### Interviews

The content analysis of the symptoms expressed in the interviews resulted in three main categories, as the symptoms revealed an engagement that was of a *bodily*, *mental/cognitive *and *emotional *nature. These main categories are presented in Table 2 along with their underlying categories, subcategories, and codes.

**Table 2:** Categorisation of all symptoms expressed in the interviews (n = 40)

**Engagement**	**Body region**	**Type of symptom**	**Symptom**	Correspondence*	n	%
Bodily	Neck	Functional	Tenseness, stiffness	1	34	85
					
			Creaking, cracking	2	6	15
					
			Weakness, tiredness, powerlessness	3–5	11	28
					
			Locking, wryneck	6	14	35
				
		Pain	Tenderness	-	4	10
					
			Gnawing, smarting	-	16	40
					
			Burning, stinging	-	10	25
					
			Cutting, pricking	-	7	18
					
			Pulsating, pounding, throbbing	-	4	10
					
			Pressure, pressuring ache	-	8	20
					
			Unspecified ache/pain	X	8	20
			
	Shoulder	Functional	Tenseness, stiffness	1	15	38
					
			Tiredness	4	3	8
				
		Pain	Tenderness	-	7	18
					
			Gnawing, smarting	-	11	28
					
			Burning, stinging	-	6	15
					
			Pressure, pressuring ache	-	4	10
					
			Unspecified ache/pain	X	9	23
			
	Shoulder blade	Functional	Tenseness, Stiffness	-	10	25
				
		Pain	Tenderness	-	4	10
					
			Gnawing, smarting	-	8	20
					
			Burning, stinging	-	2	5
					
			Unspecified ache/pain	X	8	20
			
	Rest of the body		Pain, numbness in arms	7	18	45
					
			Headache	8	22	55
					
			Ache, stiffness in jaw	9	4	10
					
			Eyes; irritated, runny, tired, blurred vision	-	3	8
					
			Throat; hoarseness, pain, cramps in larynx	10	3	8
			
	General		Dizziness	12	3	8
					
			Nausea, Vomiting	13	6	15
					
			Cold symptoms, snottiness, feebleness, feeling out of sorts	13	1	3
		
Mental/Cognitive			Fatigue	11	9	23
					
			Difficulty concentrating	14	10	25
					
			Sensitivity to sound/light	15	3	8
					
			(Burnout)		8	20
		
Emotional			Irritation, irritability	17	18	45
					
			Sadness, depression	16	6	15

*See corresponding numbers in table 6 for comparison with symptoms included in the questionnaires. (x denotes unspecified symptoms, present in both table 2 and 6, -denotes symptoms not included in any of questionnaires).

The bodily engagement consisted of the regions of the *neck*, the *shoulder*, the *shoulder blades*, the *rest of the body *and *general *engagement. The type of the symptoms of the neck, shoulder and shoulder-blade region were categorised as *functional *and as *pain*. In the neck, the *functional *symptoms were described, for example, as tenseness, stiffness, weakness, powerlessness, and lockings and/or wryneck. The experiences of *pain *were described in words as tenderness, gnawing, burning, stinging, cutting, throbbing, and pressure or pressuring ache. Unspecified ache/pain was inherent in all the pain subcategories, as some participants had trouble describing their pain experiences. In the shoulder, the *functional *symptoms were perceived as, for example, tenseness, stiffness and tiredness. Among the experiences of *pain *were descriptions of tenderness, gnawing, burning and/or stinging, and pressure or pressuring ache. It is notable that the participants seemed to refer their symptoms to the muscular region between the neck and the shoulder, rather than to the area of the shoulder joint. In the shoulder blades, the *functional *symptoms were tenseness and stiffness, while the experiences of *pain *were, for example, tenderness and gnawing pain. In the *rest of the body*, the symptoms were pain and/or numbness in the arms, headache, and ache and/or stiffness in the jaw. Symptoms from the eyes could be experienced as irritated, tired and/or runny eyes with blurry vision. Regarding the throat, there were symptoms such as hoarseness, pain, and cramp in the larynx. The participants also expressed a *general *engagement in the form of dizziness, nausea, and vomiting. In one case, even cold symptoms, such as being feeble, out of sorts, and snotty, were related to the coming and going of symptoms. Eight of the 40 participants also described symptoms of burnout, which may or may not have been related to their musculoskeletal problems.

The *mental/cognitive engagement *that the participants related to their musculoskeletal problems was experienced as symptoms as fatigue; difficulties in concentrating; and sensitivity to sound and/or light. Finally, the *emotional engagement *manifested as symptom as irritation and/or irritability; and sadness and/or depression.

### Questionnaires

#### Sources used in the development

A review of the papers that presented the different questionnaires showed that six questionnaires were modifications of low back scales (NDI, NPQ, NPDS, BQ, CNPQ, EABPS) [17,18,21,23,26]. Another, CSOQ, was developed based on literature on characteristics in neck disorders and treatment outcomes, and a consensus procedure involving health care professionals [24]. The SNQ was developed by a project group following the tradition of some earlier medical questionnaires [27]. Only EABPS and PFM had taken the experiences of those affected into consideration in the development process. The EABPS was refined by means of soliciting patients' views on the questionnaire [26]. The PFM was developed by deriving a symptom list using the experiences of 20 patients. This list was checked for "over-lapping" by a group of professionals, complemented after reviewing the literature, and then judged by the patients again (Björklund et al., In preparation). The PSFS was based on the concept of the patient generating a list of problems when answering the questionnaire [20]. A severe limitation in the PSFS' concept is that it only allows following up each individual and not comparisons between individuals.

#### Overview of all symptoms included

An initial basic content analysis was used to obtain an overview of all symptoms included in the questionnaires (Tables 3 and 4). The questionnaires differed in terms of which body regions were addressed, and the inclusion of other symptoms from the rest of the body; mental/cognitive engagement and emotional engagement also varied.

**Table 3:** Overview all symptoms included in the questionnaires

**Instrument**		**Neck/shoulder** **Symptoms**		**Symptoms from the** **rest of the body**		**Mental/Cogn**. **Engagement**	**Emotional** **engagement**
		
**Name**	**Focus**	**Pain**	**Other**	**Musculo-** **skeletal**	**Other**		
Bournemouth Questionnaire (BQ)	Neck	Neck pain				Difficulties concentrating	Anxiety (feeling tense, uptight, irritable, difficulties relaxing) Depression (feeling down-in-the-dumps, sad, in low spirits, pessimistic, unhappy)

Cervical Spine Outcome Questionnaire (CSOQ)	Neck + Shoulder arm	Neck pain + Shoulder-arm pain		Pain shoulder-arm and head ache tingling in arms or hands. Numbness, clumsiness or weakness legs	Difficulties swallowing Sleeping difficulties Felt sickly or unwell Felt low in energy or sluggish		Feeling jittery or restless Feeling anxious or tense Worry or concern about one's physical health Feeling sad, discouraged or hopeless

Core Neck Pain Questionnaire (CNPQ)	Neck + Shoulder-arm	Neck pain + Shoulder-arm pain					Feelings about spending the rest of life with these symptoms

Extended Aberdeen Back Pain Scale (EABPS)	Neck + Shoulder- arm, hand	Pain neck, back or limb Pain arm, shoulder	Weak-ness or loss of power: shoulder	Head ache Pain arm; upper arm, fore arm, or wrist/hand Weakness/loss of power; upper arm, fore arm, or wrist/hand Loss of feelings in arms	Sleeping difficulties		

Neck Disability Index (NDI)	Neck	Neck pain		Head ache	Sleeping difficulties	Affected ability to concentrate	

**Table 4:** Overview of all symptoms included in the questionnaires

**Instrument**		**Neck/shoulder symptoms**		**Symptoms from the** **rest of the body**		**Mental/Cogn**. **Engagement**	**Emotional** **engagement**
**Name**	**Focus**	**Pain**	**Other**	**Musculo- skeletal**	**Other**		

Neck Pain and Disability Scale (NPDS)	Neck	Neck pain	Neck stiffness		Sleeping difficulties	Affected ability to think or concentrate	Changed outlook on life (depression, hopelessness etc.) Affected emotions

Northwick Park Neck Pain Questionnaire (NPQ)	Neck + arms	Neck pain		Arm pain. Pins and needles in arms.	Sleeping difficulties		

Patient-Specific Functional Scale Self-Reports with Neck-Dysfunction (PSFS)	General, tested pt with neck dysfunction.	Neck pain					

Profile Fitness Mapping questionnaires (PFM)	Neck + arm-hand	Soreness Neck pain	Stiffness Tension Cracks Tiredness Weakness Lockings	Fumblingness hands Numbness Jaw trouble	Disturbance of balance Dizziness Indisposed Disturbance of swallowing Disturbance of breathing Sleeping difficulties	Disturbance of concentration Sensitivity to sound Sensitivity to light	Irritability, short tempered Depressed Stressed Anxiety Mood disturbances

Standardised Nordic Questionnaire (SNQ)	Neck + shoulder + whole loco-motor system	Ache, pain	Discomfort	Ache, pain & discomfort in: elbow, hand/wrist, upper back, low back, hips, knees, feet/ankles			

*Pain *(Tables 3 and 4) was considered as neck pain solely in BQ, NDI, NPDS, NPQ, and PFM, while pain from neck-shoulder was considered in CNPQ, CSOQ, EABPS and SNQ. *Other symptoms *from neck-shoulder were included in a few of the questionnaires, for example, stiffness (NPDS, PFM), and tension, cracking, tiredness, weakness, and locking (PFM). *Musculoskeletal symptoms *and *other symptoms *from the *rest of the body *were included in some questionnaires. The SNQ asked for pain in all regions of the body except for the head. *Musculoskeletal symptoms *were considered as symptoms in the arm/hand (CNPQ, CSOQ, EABPS, NPQ, PFM), headache (CSOQ, EABPS, NDI), and jaw trouble (PFM). *Other symptoms *from the rest of the body considered in the questionnaires were sleeping difficulties (CSOQ, EABPS, NDI, NPDS, NPQ, PFM), feeling sickly/unwell/indisposed (CSOQ, PFM), feeling low in energy or sluggish (CSOQ), dizziness or balance disturbance (PFM), difficulty swallowing (CSOQ, PFM), and difficulty breathing (PFM). *Mental/cognitive engagement *was also considered in some questionnaires as an affected ability to concentrate/think clearly (BQ, NDI, NPDS, PFM), and sensitivity to sound and light (PFM). *Emotional engagement *was considered in some questionnaires and expressed in general as affected emotions (NPDS), mood disturbances (PFM), changed outlook on life (NPDS), and feelings about spending the rest of one's life with these symptoms (CNPQ); and in specifics as depression and hopelessness (BQ, CSOQ, NPDS, PFM), anxiety and tenseness (BQ, CSOQ, PFM), stress, irritability, and short temper (BQ, PFM), and worry about physical health (CSOQ).

#### Different aspects of pain

After the initial basic content analysis, a complementary analysis of the different aspects of pain and other symptoms in the questionnaires was performed. Any eventual consideration of aspects of fluctuations (using different time perspectives) and nuances of symptoms was also reviewed, and the different response scales used in the questionnaires were examined.

The results show that the only questionnaires that took any sort of quality of pain or other neck-shoulder symptoms into consideration were the NPDS, which included neck stiffness, and PFM, which included soreness, stiffness, tension, cracking, tiredness, weakness, and locking. None of the reviewed questionnaires differentiated the sensory qualities of the neck pain, but some considered different qualities of shoulder and/or arm symptoms, such as pins and needles in the arms (NPQ), weakness or tingling in the arms or hands (CSOQ), loss of feeling, weakness, or loss of power in the arm or wrist/hand (EABP), and clumsy hands or numbness (PFM).

#### How the different aspects of pain were addressed

Pain intensity was the only aspect of pain (Table 5) that was measured by all questionnaires except for the SNQ. The SNQ instead measured prevalence of pain ever, last 12 months and seven days. Pain intensity was measured as a rating of the average level (overall/over the past week/on a typical day) (BQ, CSOQ NPDS,), at its worst (CSOQ, NPDS), and at its best (CSOQ). Pain intensity was also measured in relation to different types of activity (BQ, CSOQ, EABPS, NDI, NPDS, PFM) and rest (CSOQ, PFM). Duration was measured by EABPS (number of days in pain over the last two weeks), and SNQ (number of day in pain last 12 months), variation and duration by NPQ (continuous symptoms vs. on and off, combined with duration). Frequency and intensity were measured by PFM in combined scales ("How often?" and "How much?").

**Table 5:** How the different aspects of pain were addressed by the different questionnaires.

**Pain aspect**	**Addressed as**	**Questionnaire**
Prevalence	Ever, 12 months, 7 days	SNQ

Intensity		All questionnaires (SNQ if combined with VAS)
	
	On an average (overall/over the past week/on a typical day)	BQ, CSOQ, NPDS
	
	At its worst	CSOQ, NPDS
	
	At its best	CSOQ
	
	Related to different sorts of activity	BQ, CSOQ, EABPS, NDI, NPDS, PFM
	
	Related to rest	CSOQ, PFM

Duration	How many days in pain over the last 2 weeks?	EABPS
	
	How many days in pain last 12 months?	SNQ

Variation and duration	Continuous vs. intermittent symptoms, combined with duration	NPQ

Frequency and intensity	How often? How much?	PFM

Temporal aspects of pain	At the moment	CSOQ, NDI, NPDS, NPQ
	
	Over the last 24 hours	NPQ, PSFS
	
	Over the last 1–2 weeks	BQ, CNPQ, EABPS, SNQ
	
	Over the last 12 months	SNQ
	
	Ever	SNQ
	
	Compared to latest measurement	NPQ

Different temporal aspects of pain were considered in some of the questionnaires. Pain was asked for: -at the moment (CSOQ, NDI, NPDS, NPQ), -last 24 hours (NPQ, PSFS), -last 1–2 weeks (BQ, CNPQ, EABPS, SNQ), -last 12 months (SNQ), -ever (SNQ), and -compared to last measurement (NPQ).

#### Categorisation of all symptoms

To facilitate the comparison between the different questionnaires and to the findings from the interviews, a categorisation of all symptoms included in the questionnaires was made with respect to engagement (Table 6) that was similar to the categorisation of the content of the interviews.

**Table 6:** Categorisation of all symptoms included in the questionnaires.

**Engagement**	**Body region**	**Symptom**	Correspondence*	**Questionnaire**
Bodily	Neck only	Pain	X	BQ, NDI, NPDS, NPQ, PFM, PSFS
	
	Neck-shoulder	Pain	X	CNPQ, CSOQ, EABPS, SNQ
	
	Other neck symptoms	Stiffness	1	NPDS, PFM
		
		Tension, cracking, tiredness, weakness, locking	2–6	PFM
	
	Rest of the body	Symptoms in arm/hand	7	CNPQ, CSOQ, EABPS, NPQ, PFM, SNQ
		
		Headache	8	CSOQ, EABPS, NDI
		
		Jaw trouble	9	PFM
		
		Difficulty swallowing	10	CSOQ, PFM
		
		Difficulty breathing	-	PFM
	
	General	Sleeping difficulties	-	CSOQ, EABPS, NDI, NPDS, NPQ, PFM
		
		Feeling low in energy/sluggish	11	CSOQ
		
		Dizziness or balance disturbance	12	PFM
		
		Feeling sickly/unwell/indisposed	13	CSOQ, PFM

Mental/Cognitive		Difficulty concentrating/thinking clearly	14	BQ, NDI, NPDS, PFM
		
		Sensitivity to light/sound	15	PFM

Emotional		Affected emotions	x	NPDS
		
		Depression, hopelessness	16	BQ, CSOQ, NPDS, PFM
		
		Anxiety, tenseness	-	BQ, CSOQ, PFM
		
		Irritability, short temper	17	BQ, PFM
		
		Worry about physical health	-	CNPQ, CSOQ

* See corresponding numbers in table 2 to compare with symptoms expressed by the interviewees. (x denotes unspecified symptoms which are present in both table 2 and 6, -denotes symptoms not expressed by the interviewees).

The symptoms considered by the constructors as important to include in the questionnaires were categorised as: *bodily engagement *with pain from the *neck *or *neck-shoulder *region, *other neck symptoms*, symptoms from the *rest of the body*; and *general *symptoms; also considered were *mental/cognitive *and *emotional *engagement. None of the questionnaires covered all the symptoms included in this categorisation. PFM included 15 different symptoms, CSOQ ten, NPDS six, BQ five, EABPS and NDI four, CNPQ and NPQ three, SNQ two, and PSFS one. The distribution of symptom regions (other than neck or neck-shoulder pain) included in the different questionnaires was as follows: *other neck symptoms *in two questionnaires (NPDS, PFM), symptoms from *the rest of the body *were included in seven (CNPQ, CSOQ, EABPS, NDI, NPQ, PFM, SNQ), *general *symptoms in six (CSOQ, EABPS, NDI, NPDS, NPQ, PFM), *mental/cognitive *symptoms in four (BQ, NDI, NPDS, PFM), and *emotional *symptoms in five (BQ, CNPQ, CSOQ, NPDS PFM). PFM had the best coverage, with five aspects of symptoms included (beside the neck or neck-shoulder pain). NPDS included four aspects, NDI and CSOQ three, BQ, CNPQ, EABPS, NPQ and SNQ included one aspect, while PSFS included only pain from the neck region.

#### Correspondence between questionnaires and interviews

We found both similarities and differences between the specific symptoms described in the interviews and the symptoms included in the questionnaires. It is important to note that the following comparisons were made to the questionnaires taken all together, as a whole; when comparing to each of the questionnaires separately, the correspondence in most cases was low. The following comparisons are summarised under correspondence in Tables 2 and 6, in which the numbers after each symptom correspond to the occurrence of that symptom in the other table, and a dash (-) indicates that the symptom was not found in the data presented in the other table.

All functional symptoms from the neck derived from the interviews were found in the questionnaires as tenseness, stiffness, weakness, tiredness, powerlessness, cracking, and locking. According to the pain experiences the interviews revealed a qualitative differentiation which was not considered in the questionnaires. The functional symptoms from the shoulder described in the interviews might have been considered in the questionnaires as tenseness, stiffness, and tiredness in neck-shoulder. The differentiated pain symptoms from the shoulder described in the interviews were not considered in the questionnaires. In the interviews, some functional symptoms and pain symptoms from the shoulder blade region were described, while no such symptoms were included in the questionnaires. For the rest of the body, symptoms as pain and/or numbness in arms, headache, ache and/or stiffness in the jaw and throat symptoms were present in both interviews and questionnaires. Eye symptoms, which were not considered in the questionnaires, were mentioned by a few of the participants. General engagement was present in the interviews as dizziness, nausea, vomiting, cold symptoms, feebleness, and feeling out of sorts. This was interpreted as corresponding to feeling sickly/unwell/indisposed. Mental/cognitive engagement, expressed as fatigue, difficulty concentrating, and sensitivity to sound and light, was present in both interviews and questionnaires. In the interviews, eight people described symptoms of burnout, which might be a side finding as the participants did not relate those symptoms directly to their neck-shoulder disorder but may be worth further investigation. In terms of emotional engagement, irritation and irritability, and sadness and depression, were considered in both interviews and questionnaires.

Once again, it should be noted that these comparisons include the questionnaires as a group, and not separately. No single questionnaire covered all symptoms from the interviews. The PFM was the most comprehensive of the questionnaires (Table 6).

Conversely, the questionnaires did cover a couple of aspects that are not presented in the categorisation of the interviews. Firstly, sleeping difficulties, which were not interpreted as a symptom, per se, as the participants attributed their sleeping difficulties to their pain and other symptoms. Therefore it is not included in the categorisation presented in Table 2, even though it was present in the interviews. Nevertheless, as we know that sleeping problems have an adverse effect on health, it seems sound to also include in questionnaires. Secondly, breathing difficulties, which were not mentioned at all in the interviews.

#### Summary of results

• The narratives of men and women with neck-shoulder disorders revealed a variety of symptoms related to such disorders.

• These symptoms showed a bodily, mental/cognitive, and emotional engagement, and included more general and more severe symptoms than are usually found in neck-shoulder questionnaires.

• Few neck-shoulder questionnaires were developed using the experiences of those affected.

• Most neck-shoulder questionnaires covered only a minority of the symptoms presented by people with neck-shoulder disorders.

• Mental/cognitive and emotional engagement, presented as significant symptoms by the participants, was often overlooked in the questionnaires.

• The fluctuations of symptoms were usually not taken into consideration.

• The nuances of symptoms were rarely considered.

## Discussion

There is increasing agreement regarding the value of taking the experiences of those affected into consideration in the development of health status measurements [6-9], but this is still a rare practice in the area of musculoskeletal disorders. The usual sources of items in questionnaires are published literature, health professionals, and existing questionnaires [6]; this was also the case with the ten questionnaires reviewed in the present study. Six out of ten of the reviewed questionnaires were modified low back questionnaires. Only two questionnaires (EABPS, PFM) had used the opinions of those affected in the development process. The development process of EABPS used opinions on whether the questions were unclear, badly phrased, annoying, or unnecessary, or if any issue was omitted; this is a procedure which mostly relates to the face validity of the questionnaire. On the other hand, the PFM used symptom experiences to decide which items to include, a method which seems more likely to enhance the content validity. The PFM, which used the symptom experiences of 20 people with neck-shoulder problem in its development, seems to better reflect the significant aspects, as it had the greatest correspondence to the content of the interviews performed in our study. A number of researchers have emphasised the need to develop and choose instruments that fit the target group and the purpose of the evaluations [8,15]. Many neck-shoulder questionnaires are modifications of low back scales. A better approach might be to consider the content and items of several existing neck-shoulder questionnaires and the experiences of people with the disorder in order to increase the possibility of reflecting significant aspects of the disorder and thereby improve the content validity of the questionnaire.

Most of the analysed neck-shoulder questionnaires covered only a minority of the symptoms presented by people with neck-shoulder problems. The Initiative on Method, Measurement, and Pain Assessment in Clinical Trials (IMMPACT) have recommended some core outcome domains to consider when designing clinical pain trials, i.e. pain, physical functioning, emotional functioning, global improvement, satisfaction with treatment, symptoms and adverse events [10,11]. In terms of pain and functional symptoms, we noted that none of the reviewed questionnaires included symptoms from the shoulder blade region, a region mentioned by 25% of the participants. Emotional functioning is also often overlooked. The questionnaires that considered several aspects of emotional engagement were BQ, CSOQ, and PFM; the others either asked diffusely or not at all. The IMMPACT group has presented the result of a survey among people with pain, regarding their view of important outcome domains for chronic pain clinical trials [29]. The aspects that were judged to be the most important, in addition to pain reduction, were enjoyment of life, emotional well-being, fatigue, weakness and sleep-related problems. These findings are in line with the results of the present study and point at the importance of other aspects than just pain and physical functioning as outcome measures. Mental/cognitive engagement is not one of the suggested outcome domains of Dworkin and collegaues [10,11]. However, it was clearly present as difficulty concentrating/thinking in four of the analysed questionnaires (BQ, NDI, NPDS, PFM), and as sensitivity to sound and light in one (PFM). The mental/cognitive engagement mentioned in the interviews included difficulty concentrating and sensitivity to sound/light, but also fatigue, which was experienced by about one-fourth of the participants. To increase the content validity of a neck-specific questionnaire, these aspects should also be included.

The fluctuation of symptoms is usually not taken into consideration in questionnaires, and similarly the nuances of symptoms are not considered. These aspects seem to be of great importance in relation to musculoskeletal disorders, as a great variability of the course of the disorder including a variety of symptoms is inherent in such conditions [30-33]. An exploratory study of the experience of bodily illness among people with chronic neck-shoulder problems [14] describe a course of the disorder which is characterised by uncontrollable fluctuations. On a daily basis, most of the participants experienced constant discomfort; on top of this they reported intermittent fluctuations in symptom intensity and periods of peak intensity. By taking different quality and temporal aspects into consideration, as recommended by Dworkin and colleagues [10], it is possible to better capture the patient's symptom experience and thereby increase the possibility of identifying treatments effective for certain aspects of symptoms, and also enable better evaluations.

Another of the six domains to consider in clinical pain trials comprises adverse events [10]. In the above mentioned interview study regarding the disease course of chronic neck-shoulder problems [14], moments of consuming intensity were described, an aspect which might be included in this domain. Some of the participants in the previous study and in the present study described symptoms of a general bodily engagement with different symptoms during intense periods. This engagement included symptoms from eyes and throat, dizziness, vomiting, feebleness, and feeling out of sorts. This intensity aspect of the variable symptoms is often not taken into consideration, and could be an area worth studying further.

The participants were able to distinguish a variety of different qualities or nuances of symptoms related to the different regions. Different approaches to facilitate the description and measurement of pain have been considered in general pain questionnaires, such as the McGill Pain Questionnaire (MPQ) [34,35]. Melzack [34,35] identified a variety of pain descriptors which he categorised into four major groups; sensory, affective, evaluative, and miscellaneous. Different temporal aspects were also considered, such as whether the pain was experienced as rhythmic, periodic, intermittent, continuous, steady, or constant. Taking such an approach to musculoskeletal disorders could increase the possibility of capturing the fluctuations of symptoms, thus potentially increasing both the criterion-related validity (the ability to predict some criterion variable, such as the course of the underlying disease) and the responsiveness (the ability to detect small but important clinical changes) [15].

To develop a condition-specific questionnaire it is important to decide on the specific symptoms for the condition. A good way to do this is by using the symptom experiences of those affected, combined with relevant research and professional knowledge. The use of a combination of insider/outsider perspectives and qualitative/quantitative methods is needed if we are to successfully develop a questionnaire that captures the spectrum of symptoms experienced by individuals suffering from neck-shoulder pain in order to alleviate the suffering of those affected.

## Conclusion

Even though musculoskeletal epidemiology over the last decades has recognized the multidimensionality of the disorders, there still remain some issues related to neck-shoulder disorders (as well as to musculoskeletal disorders in general). One is that pain and disability often are the only aspects that are considered as relevant measures. Another is the use of a variety of combinations of different questionnaires measuring different aspects, and/or a selection of questions from these questionnaires. This, combined with different definitions of the disorders and included body regions, makes it difficult or impossible to accurately depict the problem, evaluate results and to make comparisons between studies. As it is reasonable to suppose that there are considerable differences in the multidimensionality between different musculoskeletal disorders, focus on measurement of neck-shoulder pain is needed.

Although there is increasing agreement regarding the value of taking the experiences of those affected into consideration in the development of health status measurements, this is still a rare practice in the area of neck-shoulder pain. The present study shows that few of the analysed questionnaires used were developed using the experiences of people affected with the disorders. Instead, many of the questionnaires were modifications of low back scales. A better approach might be to consider the content and items of several existing neck-shoulder questionnaires and the experiences of people with the disorder in order to increase the possibility of reflecting significant aspects of the disorder and thereby improve the content validity of the questionnaire.

A variety of symptoms were expressed by the interviewees. The symptom experiences revealed a bodily, mental/cognitive, and emotional engagement, and included more severe symptoms than are usually related to neck-shoulder disorders. The participants were also able to distinguish a variety of different qualities or nuances of symptoms related to the different regions, as well as fluctuations of symptoms.

The correspondence between the separate questionnaires and the experiences of those affected was low; most of the analysed neck-shoulder questionnaires covered only a minority of the symptoms presented by people with neck-shoulder problems. Mental and emotional engagement was often overlooked in the questionnaires, and fluctuations and nuances of symptoms were rarely considered. These aspects seem to be of great importance in relation to musculoskeletal disorders, as a great variability of the course of the disorder including a variety of symptoms is inherent in such conditions.

The present study points at the importance of other aspects than just pain and physical functioning as clinical trial outcome measures related to neck-shoulder disorders. By also taking different quality and temporal aspects into consideration, it is possible to better capture the patient's symptom experience and thereby increase the possibility of identifying treatments effective for certain aspects of symptoms, and also enable better evaluations.

## Competing interests

The authors declare that they have no competing interests.

## Authors' contributions

BW, CB and MD participated in the design of the study. BW performed the data collection. BW and CB were responsible for the analysis of data. All authors (BW, MB, CB and MD) participated in the drafting of the manuscript, and the revision of the draft. All authors (BW, MB, CB and MD) read and approved the final manuscript.

## Pre-publication history

The pre-publication history for this paper can be accessed here:


